# Evaluation of a Novel Mechanical Device for the Production of Microfragmented Adipose Tissue for Veterinary Regenerative Medicine: A Proof-of-Concept

**DOI:** 10.3390/ijms252111854

**Published:** 2024-11-04

**Authors:** Priscilla Berni, Valentina Andreoli, Virna Conti, Roberto Ramoni, Giuseppina Basini, Gabriele Scattini, Luisa Pascucci, Martina Pellegrini, Maurizio Del Bue, Gian Paolo Squassino, Francesca Paino, Augusto Pessina, Giulio Alessandri, Paolo Pirazzoli, Antonio Bosetto, Stefano Grolli

**Affiliations:** 1 Department of Veterinary Medical Science, University of Parma, 43121 Parma, Italy; valentina.andreoli@unipr.it (V.A.); virna.conti@unipr.it (V.C.); roberto.ramoni@unipr.it (R.R.); giuseppina.basini@unipr.it (G.B.); 2 Department of Veterinary Medicine, University of Perugia, 06123 Perugia, Italy; gabriele.scattini@unipg.it (G.S.); luisa.pascucci@unipg.it (L.P.); 3 Istituto Zooprofilattico Sperimentale dell’Umbria e delle Marche “Togo Rosati”, 06126 Perugia, Italy; m.pellegrini@izsum.it; 4 Independent Researcher, 43121 Parma, Italy; mauriziodelbue@gmail.com; 5 Studio Tecnico Veterinario, 14100 Asti, Italy; squassino@yahoo.it; 6 CRC StaMeTec, Department of Biomedical, Surgical and Dental Sciences, University of Milan, 20122 Milan, Italy; francesca.paino@unimi.it (F.P.); augusto.pessina@unimi.it (A.P.); cisiamo2@yahoo.com (G.A.); 7 Hydra srl, 41037 Mirandola, Italy; paolo.pirazzoli@hydra-srl.com (P.P.); antonio.bosetto@hydra-srl.com (A.B.)

**Keywords:** regenerative medicine, mesenchymal stromal cells, microfragmented adipose tissue, dog, veterinary medicine

## Abstract

Therapies based on mesenchymal stromal cells (MSCs) have become one of the most significant advancements in veterinary regenerative medicine. The isolation of MSCs is usually performed by enzymatic digestion and requires variable times for cell expansion. In addition, these procedures need to be performed in specialized laboratory facilities. An alternative approach to in vitro-expanded MSC therapy is the use of microfragmented adipose tissue (microfat), which is a rich source of cells and growth factors from the stromal vascular fraction. Recent clinical studies support its safety and efficacy in the treatment of musculoskeletal disorders and wound healing. The aim of the present work was to characterize the microfragmented adipose tissue obtained by a new mechanical device, which provides sterile tissue that is ready for use in the clinic by the veterinarian, avoiding the need for specialized laboratory facilities. Microfat-derived MSCs were compared with enzymatically isolated MSCs in terms of their phenotypic characterization, growth rate and differentiation potential. Conditioned medium derived from microfat culture was evaluated for its ability to promote MSC vitality. No differences were observed between MSCs obtained through mechanical fragmentation and those derived from collagenase digestion of adipose tissue, suggesting that the device could serve as a practical source of microfragmented adipose tissue for use in veterinary clinics.

## 1. Introduction

Veterinary regenerative medicine is receiving ever-wider interest in clinical practice. Initially developed around musculoskeletal disorders (tendon, ligament and joint diseases) [1,2], mesenchymal stromal cell (MSC)-based therapies have recently expanded their potential applications. The focus is now broadening to encompass kidney, liver, heart, respiratory and digestive system diseases [3]. MSCs are also actively investigated for the treatment of inflammatory systemic diseases, immune-mediated disorders, chronic degenerative diseases [4,5,6] and for the treatment of drug-resistant infections [7]. Although initially used as a stem cell population capable of integrating and differentiating into the damaged tissue, the demonstration that the majority of the biological effects of MSCs are driven by the release of a complex secretome has transformed the paradigm of their use [8,9].

At the same time, the range of cell preparations available for clinical use has gradually expanded. Although the use of isolated and in vitro-expanded autologous MSCs remains the cornerstone of these therapies, allogeneic MSC preparations [10], adipose-tissue-derived stromal vascular fraction (SVF) and microfragmented fat (microfat) have found their place in clinical practice [11,12,13]. Although the current use of allogeneic cells is still controversial due to safety and efficacy issues, which have been largely discussed in the literature [10,14], a relevant number of investigations suggest that allogeneic MSCs may represent a safe alternative to autologous MSCs, and they also provide practical advantages, such as the ability to bank the cells and to reduce the time to treatment [2,10,14]. Despite these key aspects, the safety of allogeneic MSCs must be established before clinical use through the use of careful controls as a good practice to minimize the risk of donor-derived infectious diseases and potentially dangerous immune responses [2,14,15]. National regulatory authorities, such as the USDA and EMA, insist on this safety aspect, although a standard regulatory pathway for allogeneic MSC use does not yet exist [6,16,17,18]. However, the EMA has recently approved the marketing of allogeneic and xenogeneic MSC-based products based on their safety and efficacy data [19,20]. In particular, DogStem is a xenogenic cell treatment consisting of equine umbilical cord MSCs approved for the treatment of canine osteoarthritis [21] while Arti-cell Forte, which consists of chondrogenic-induced equine allogeneic peripheral-blood-derived mesenchymal stem cells, has been approved for the treatment of non-septic joint inflammation in horses [22].

Among the different therapeutic approaches based on MSCs, the use of SVF and microfragmented fat is an attractive alternative for the veterinary surgeon due to several practical advantages. SVF and microfat allow ready-to-use autologous cell preparations to be obtained, avoiding cell manipulation and minimizing external microbiological contamination. In addition, they represent ready-to-use products that avoid the time-consuming delays that characterize the in vitro expansion of autologous cells [2,23]. Although these preparations differ in composition and therapeutic potential, they can be processed and used by the veterinarian in a single therapeutic session using commercial kits. Usually, a short time is required from tissue collection to product application. A specialized laboratory is not required for the expansion of the cells, nor are there long waiting times associated with the cell preparation.

Adipose-tissue-derived products have extensive scientific literature supporting their clinical use in musculoskeletal conditions, wounds and skin lesions [24,25]. Among them, microfragmented fat (referred to as microfat or nanofat, depending on the size of the fragments obtained) is the best-described in the literature and the most widely used [26,27]. Adipose tissue is a rich source of MSCs. When used to produce microfat, it provides a complex therapeutic preparation in which numerous other cell types (stromal progenitor cells, fibroblasts, endothelial cells, pericytes and blood-derived cells) are present. In addition, the presence of extracellular matrix components (collagens, fibronectin and laminin) are present provide a 3D scaffold for the aforementioned cells. Cells and matrices are usually maintained in a tissue-like structural and functional organization [28,29]. Furthermore, the interest in microfragmented fat has recently been extended to cancer chemotherapy, as these preparations provide a scaffold capable of delivering and releasing anticancer drugs [30,31], thus expanding their therapeutic potential when associated with microfat preparation.

The aim of the present work was to develop a mechanical device (T-Grinder™) that applies minimal shear stress and a blade-based cutting system to prepare microfragmented tissue that is ready for clinical use in a short time from fat boluses of variable dimensions. The device, which has been developed according to a prototype that can be easily adapted to treat different amounts of tissue (4–20 g), has been tested on perivisceral adipose tissue sampled from dogs. The biological properties of the microfragmented fat were evaluated by an analysis of cell viability and growth, while phenotypic characterization was performed by flow cytometry, and trilineage differentiation and gene expression was analyzed by real-time PCR. The data obtained demonstrate the potential of using this instrument to prepare microfragmented fat from which MSCs can be isolated and expanded in vitro while maintaining characteristics identical to MSCs obtained through traditional enzymatic tissue dissociation methods.

To assess the viability and generic equivalence of the mechanical processing method versus standard enzymatic digestion of the initial biological material—canine adipose tissue—and to draw conclusions concerning the biological activity and characteristics of the two groups of cell cultures, only a limited statistical donors’ population was required, while a larger test repetition number was planned and used to confirm and validate the repeatability and reliability of the obtained results at a cellular level in the laboratory.

A much larger number of cases should constitute the basis for subsequent controlled studies that are devoted to assessing the clinical outcome of this one-step regenerative medicine therapeutic approach, made possible by the mechanical microfragmentation of adipose tissue in an operating theater, versus the isolation, expansion and delayed administration of allogenic biological material in clinical cases related to specific pathologies, using a controlled and statistically significant selected cohort of patients.

## 2. Results

### 2.1. Microfat Culture

**Figure 1:**
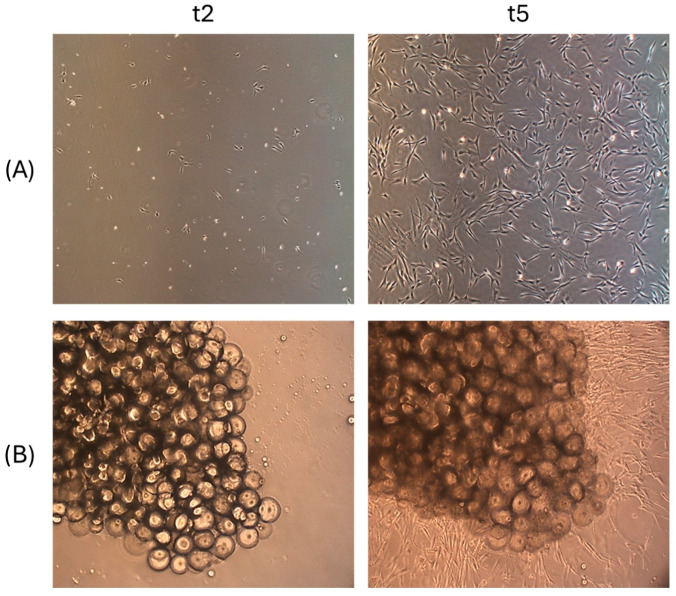
(**A**) Ad-MSCs after 2 (t2) and 5 (t5) days of 2D culture in 25 cm^2^ flasks (10×). (**B**) Ad-MSCs isolated from microfragments in a 3D matrix at 2 (t2) and 5 (t5) days of culture.

The mechanical device used to process fat tissue samples allowed for the isolation of cells capable of adhering to a plastic surface and maintaining a fibroblast-like shape when cultured in a conventional 2D environment (Figure 1A). When cultured in a 3D fibrin matrix, 100% of the non-digested fat microfragments demonstrated an active outgrowth of cells (Figure 1B) that gradually expanded inside the matrix itself, as already observed for human adipose tissue fragments [26,32].

### 2.2. Cell Count

**Figure 2:**
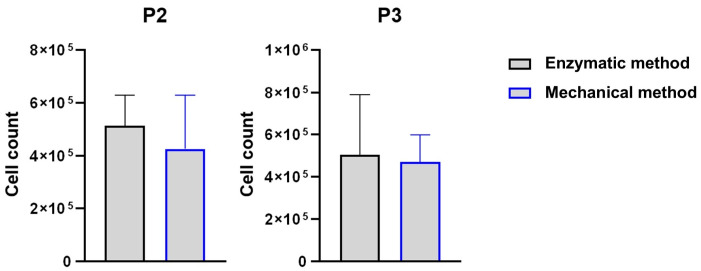
Average number of cells obtained using the two preparation methods (mechanical processing and enzymatic digestion) at P2 and P3 (*n* = 4).

The cell count was performed at P1 when the cells reached about 80% confluence. For both isolation methods, i.e., microfragmentation and enzymatic dissociation, the MSCs were then expanded to P2 and P3 in 6-well plates and counted when 80% confluent (Figure 2). In detail, the total cell number was 1.74 ± 0.5 × 10^6^ at P1, 4.25 ± 2.03 × 10^5^ at P2 and 5.03 ± 0.28 × 10^5^ at P3 for the grinder population and 1.65 ± 0.7 × 10^6^ at P1, 5.13 ± 1.16 × 10^5^ at P2 and 4.71 ± 0.12 × 10^5^ at P3 for the control population. No statistically significant differences were observed between the two methods.

**Figure 3:**
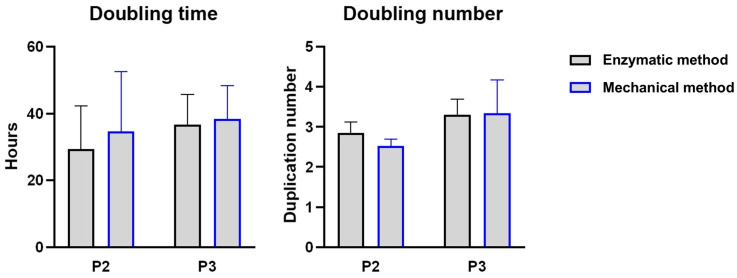
Bar graphs illustrating the cell-doubling time (left) and cell-doubling number (right) of cells obtained using the two preparation methods (mechanical processing and enzymatic digestion) at P2 and P3 (*n* = 4). No statistically significant differences are observed between the two methods.

The cell-doubling time and cell-doubling number were also not significantly different at both P2 and P3, as shown in Figure 3.

### 2.3. Histological Analysis

**Figure 4:**
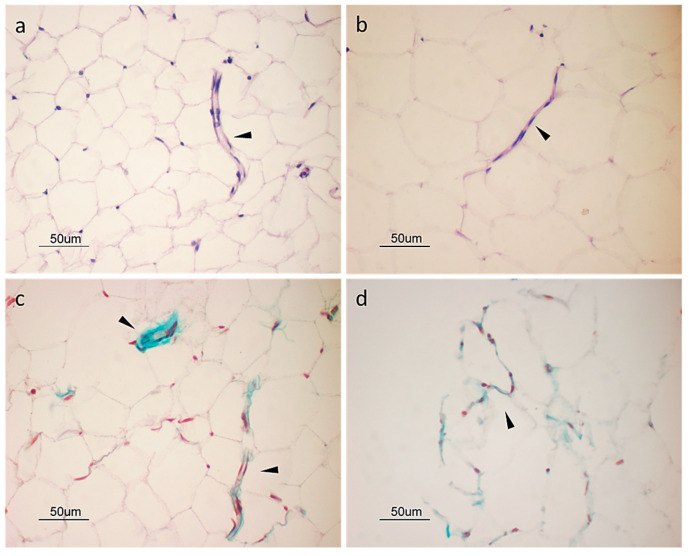
Histological staining of adipose tissue before (**a**,**c**) and after (**b**,**d**) mechanical processing. Five-micron-thin sections were stained with hematoxylin and eosin (**a**,**b**) and by Masson trichrome staining (**c**,**d**). Stromal vascular fraction elements are indicated by arrowheads.

Under histological analysis, the adipose tissue displayed classical features. In particular, the adipocytes were organized in “honeycomb” structures and small vessels and a small amount of extracellular matrix were observed (Figure 4a,c). Adipose tissue processed by mechanical methods showed minor changes in tissue structure: in particular, the adipocytes appeared larger and more variable in shape than the original cells, while vessels and other stromal elements displayed characteristics similar to those of the original tissue (Figure 4b,d).

### 2.4. Ad-MSCs Trilineage Differentiation

**Figure 5:**
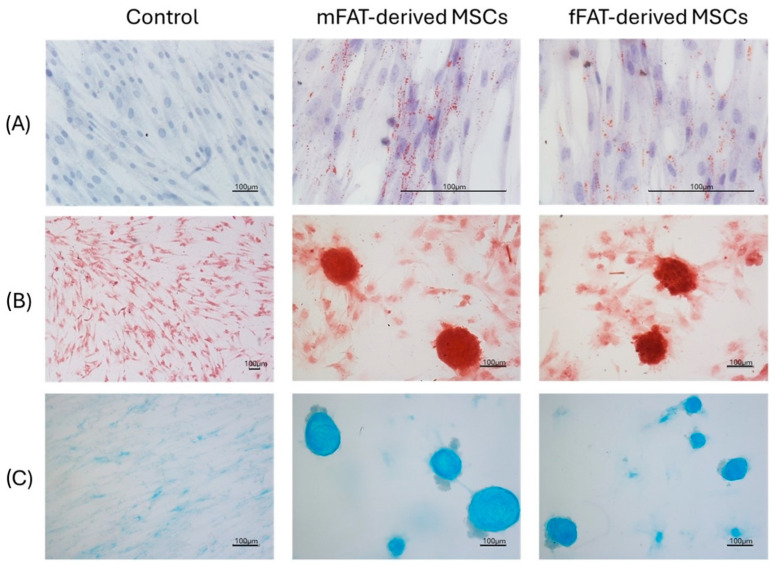
Adipogenic (**A**), osteogenic (**B**) and chondrogenic (**C**) differentiation of mFAT-derived MSCs compared with fFAT-derived MSCs at P3. Unstimulated cells were used as a control group.

Ad-MSCs isolated from both methods were able to undergo adipogenic, osteogenic and chondrogenic differentiation (Figure 5). Following stimulation with adipogenic differentiation media, both cell cultures demonstrated the presence of Red Oil O-stained cells. Osteogenic differentiation was demonstrated by positive Alizarin Red staining in both microfragmented adipose tissue (mFAT) and control enzyme-digested adipose tissue (fFAT) cultures treated with osteogenic medium. The same trend was observed for chondrogenic differentiation. Unstimulated control cultures were negative for all the specific stains tested.

### 2.5. Gene Expression Analysis

**Figure 6:**
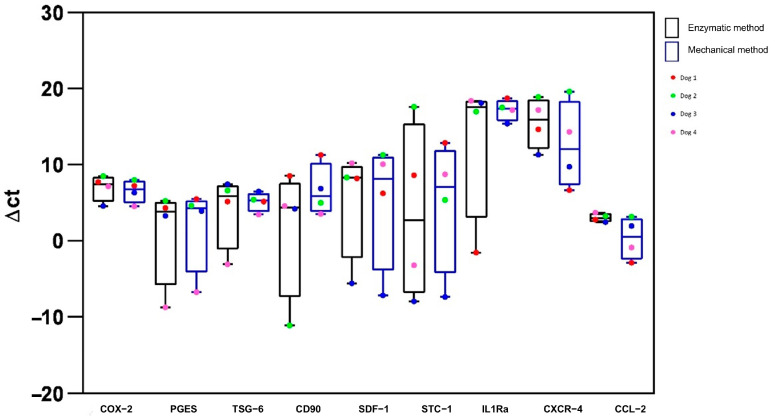
Box plot (median, quartiles) representing the ΔCt values of COX-2, PGES, TSG-6, CD90, SDF-1, STC-1, IL1Ra, CXCR-4 and CCL-2 in Ad-MSCs at P3 prepared using the two methods (enzymatic and mechanical). No significative differences were found between the two preparations.

The expression of a panel of genes involved in the immunomodulatory properties of MSCs was assessed by real-time PCR quantitative analysis. No statistically significant differences were observed for the targeted genes, suggesting no differences between the two preparation methods (Figure 6).

### 2.6. Flow Cytometry

**Figure 7:**
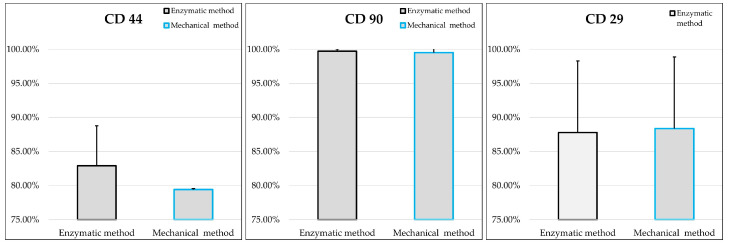
Mean values ± standard deviations of P3 cells positive for CD44, CD90 and CD29 that were obtained by the enzymatic (grey) or mechanical (blue) method. No statistically significant differences were found between the two groups (*n* = 3).

The two populations of MSCs at P3 were compared by evaluating the expression of positive (CD29, CD90, CD44) and negative (CD45, CD14, MHCII) surface markers, as suggested by the ISCT [29]. Percentages of positive cells for the tested markers are shown in Table 1. In detail, MSCs derived from both the mechanical and the enzymatic procedure showed a strong positivity for CD29 and CD90 and CD44 markers (Figure 7; Table 1).

**Figure 8:**
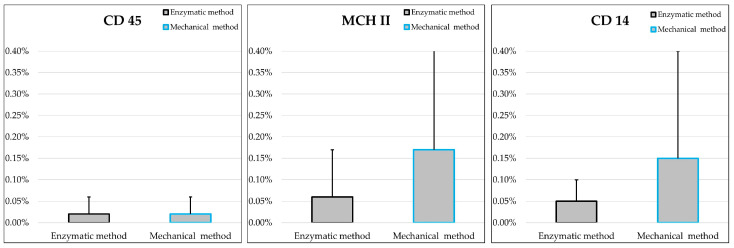
Mean values ± standard deviations of P3 cells positive for CD45, MHC II and CD14 that were obtained by the two methods (enzymatic and mechanical). No statistically significant differences were found between the two groups (*n* = 3).

The two populations of MSCs were negative for CD14, CD45 and MHCII, with values below the 2% threshold suggested by the ISCT (Figure 8; Table 1).

**Figure 9:**
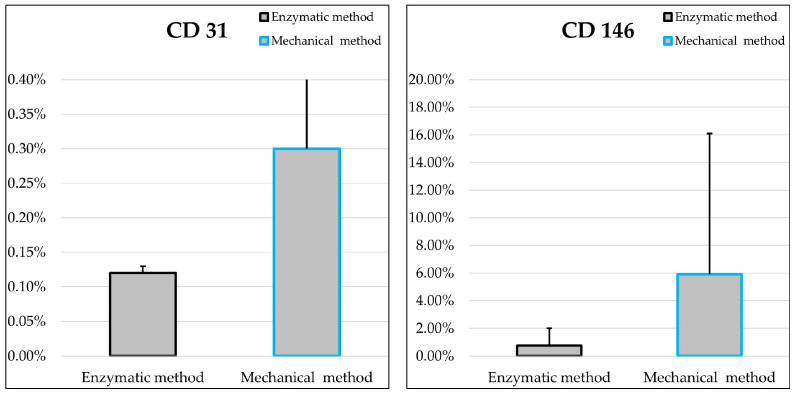
Mean values ± standard deviations of P3 cells positive for CD31 and CD146 that were obtained by the two methods (enzymatic and mechanical). No statistically significant differences were found between the two groups (*n* = 3).

MSCs prepared by the two procedures demonstrated low expression of the endothelial marker CD31, while the percentage of CD146-positive cells was higher in cells derived from the mechanical treatment (Figure 9; Table 1). CD146 is considered a marker of endothelial cells and pericytes [29], but its expression has been also reported in human and equine MSCs [33,34,35].

Percentages and standard deviations of cells positive for the tested markers are reported in Table 1. No statistically significant differences were found comparing the enzymatic and the mechanical processing methods.

### 2.7. Effects of Microfat-Conditioned Medium on Cell Vitality

**Figure 10:**
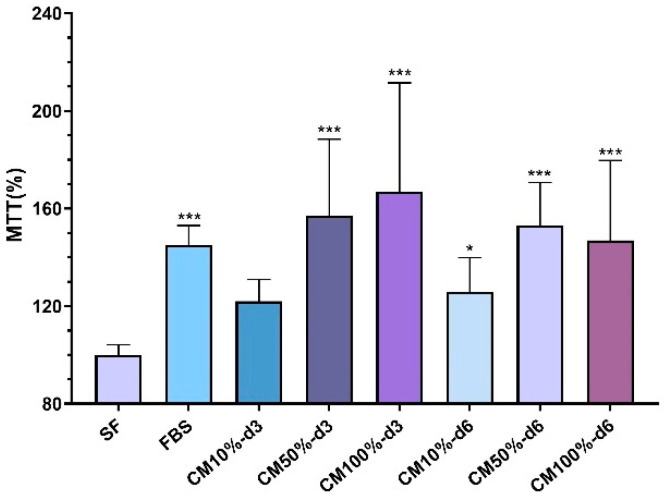
Effect of microfat-conditioned medium (CM) on canine MSCs’ metabolic activity as measured by the MTT assay. Data are expressed as a percentage of the activity of control cultures maintained in serum-free DMEM (SF). FBS: 10% FBS-DMEM; CM10%, CM50%, CM100%: percentage of CM in the final volume of serum-free DMEM; d3 = CM collected on day 3; d6: CM collected on day 6. * *p* < 0.05; *** *p* < 0.001.

To evaluate the ability of the conditioned medium prepared with T-Grinder^TM^ microfat to support the metabolic activity of canine MSCs, different dilutions of CM collected after 3 and 6 days of fragment culture in serum-free DMEM were compared by the MTT assay with serum-free DMEM and DMEM supplemented with 10% (*v*/*v*) FBS. The MTT assay showed that microfat-derived CM had a positive effect on cell viability compared with serum-free medium (Figure 10). The difference was statistically significant (*p* < 0.001) for every time point evaluated for the 50% (*v*/*v*) and 100% CM treatments. When the CM was diluted to 10% *v*/*v* with serum-free medium, there was a statistically significant improvement in cell metabolic activity on day 6 (*p* < 0.01), but no difference was observed on day 3. CM treatments maintained a comparable stimulus to that obtained with 10% FBS medium, thus confirming the potential of microfat CM to maintain the vitality of canine MSCs.

## 3. Discussion

Regenerative medicine is becoming increasingly popular in veterinary clinical practice [6]. However, the requirement for autologous MSCs represents a critical limit to the diffusion of cell therapies [2,10,15]. Given the complexities associated with the administration of autologous cell therapies, the availability of a “minimally manipulated” biological product is a valid alternative to the use of in vitro-expanded cells. The EMA and the FDA recognize that ”minimal manipulation” is applied to a cell therapy protocol when tissues are treated by non-enzymatic mechanical methods and the preparations do not undergo in vitro expansion and biological and physiological modifications. The use of minimal mechanical procedures allows for the delivery of cellular elements, cellular matrix components and soluble biomolecules that can contribute to tissue regeneration within the same surgical intervention [36,37]. In this field, biological therapeutics derived from adipose tissue are of great interest given the relative simplicity of obtaining adipose tissue in veterinary patients, especially in canine species [2].

MSCs can be isolated and expanded from various tissue sources, including adipose tissue and bone marrow [6]. However, the process is time consuming and expensive, requiring sophisticated multi-step procedures, sterility testing and assessment of the biological activity of the preparations. Consequently, this therapeutic intervention requires a time delay for the preparation of a suitable cell population. Adipose tissue (AT) is probably the preferred source of canine MSCs due to its more accessible collection and higher cell yield compared with bone marrow. MSCs can be obtained from subcutaneous or perivisceral adipose tissue samples. Bahamondes et al. [38] suggested that adipose tissue harvesting is easily successful for intra-abdominal sources (omental or peri-ovarian tissue). On the other hand, the collection of subcutaneous AT depends on the patient’s size and body mass index. The same group of investigators showed that the cell yield is higher in omental tissue, while other characteristics of the cell (cell proliferation, senescence, expression of a range of growth factors and cytokines) are conserved in comparison with subcutaneous adipose-tissue-derived MSCs. Hendawy et al. [39] suggested that in dogs, a peri-ovarian harvest site is the best for AT sampling in terms of MSC yield. Based on these considerations, we evaluated the feasibility of obtaining microfragmented fat from perivisceral adipose tissue.

Devices that are capable of microfragmenting adipose tissue for the preparation of a minimally manipulated specimen that can be applied in the same surgical session may prove to be beneficial in clinical practice, as evidenced by recent human clinical trials [29], particularly for therapeutic applications in musculoskeletal disorders. Positive clinical outcomes following microfat therapy have also recently been highlighted in veterinary medicine, thus demonstrating its potential in veterinary clinical practice. Zeira et al. treated 130 dogs affected by OA with microfat and demonstrated an improvement in the orthopedic scores of the patients without local or systemic adverse effects [13]. Pennasilico et al. used microfragmented adipose tissue in 10 dogs undergoing tibial plateau-leveling osteotomy (TPLO) and found that the treatment could accelerate bone healing [24]. No adverse effects were reported. Interestingly, a case report by Zeira et al. [30] suggests that microfragmented fat loaded with an anti-cancer drug can be used as a carrier to deliver chemotherapy to the tumor site.

The device we describe here, T-Grinder™, is designed to produce microfat from samples ranging from 6 to 20 g of perivisceral adipose tissue. However, the characteristics of the device also lend themselves to the use of subcutaneous adipose tissue. The procedure involves harvesting the sample in small fragments (approximately 1–1.5 g each) during the surgical procedure, transferring them directly into the sterile device and processing them with a single passage through the manually operated blade system. Finally, the microfragmented fat is collected in a luer lock syringe attached to the device (see Supplementary Materials Video S1). The procedure does not require the addition of saline washing solution, and the product collected in the collecting syringe can be immediately applied to the patient. Although mechanical processing partially alters the structure of the fat tissue, the stromal structure of the tissue, such as blood vessels and interstitial cells, is preserved (Figure 4), as is the viability of the fragments (Figure 1). Similar results were reported by Eigenberger et al., who concluded that short-term mechanical stress does not lead to the sustained modification of the biological features of fat tissue [40].

To confirm the feasibility of the procedure and to evaluate the performance of the T-Grinder™, we compared the MSCs obtained from microfragmented AT with MSCs prepared with the traditional collagenase-based MSC method. The comparison was based on the following parameters: the number of MSCs obtained per gram of tissue, the growth capacity (from P1 to P3), phenotypic characterization by flow cytometry and the capacity for differentiation. Furthermore, cell populations were compared by evaluating the expression of a panel of genes whose roles are important in the functional characterization of MSCs [41]. The results of the present investigation support the ability to prepare biologically active microfat preparations by a single passage through the instruments, aimed at reducing the shear stress generated by the blade system and extrusion through the sieve. Cells derived from the grinder were confirmed as MSCs by immunophenotyping. There were no significant differences between the microfat and collagenase-derived cells in the expression of the typical MSC-positive markers CD90 and CD29. Lower expression of CD44 was observed in the microfat-derived cells (79.41 ± 10.13), although this was not statistically significant. In any case, the average percentage of CD44-positive cells is within the range reported by other studies for adipose-derived canine MSCs [39,42]. As for the negative surface markers CD45, CD14 and MHCII, their expression was less than 2%, as suggested by the ISCT. The endothelial marker CD31 was not expressed in both MSC populations, whereas a higher, although not statistically significant, expression was observed for CD146. Although CD146 is considered an endothelial and pericyte cell marker, its expression has been reported in human and equine MSCs and may be related to their multipotency [33,34,35]. Recently, Bikorimana et al. [43] suggested that MSC populations enriched in CD146^+^ cells exhibit an enhanced immunosuppressive profile. Interestingly, the population enriched in CD146^+^ cells showed a lower CD44 expression level. Our data support the hypothesis that the mechanical device can indeed provide a population of MSCs enriched in CD146^+^ cells with a lower expression of CD44, which is worth further study in terms of its immune-modulating properties. The cell-doubling time and cell-doubling number confirmed that the growth rate of MSCs is maintained at the same level in the two different MSC preparations. Osteogenic, chondrogenic and adipogenic differentiation is also maintained in cells derived from microfragmentation. These data support the hypothesis that cell populations essentially equivalent to MSCs can be obtained using T-Grinder™, generating adipose tissue fragments where the proliferation of resident MSCs is preserved after the mechanical processing.

To assess the viability of microfragments obtained by means of the T-Grinder^TM^, in addition to the isolation and characterization of MSCs, the ability to generate an outgrowth of cells when the fragments were cultured in a 3D environment was evaluated. Furthermore, the effect of microfragment-conditioned medium on the metabolic activity of canine MSCs was investigated. The capacity of the microfragments to generate outgrowth cells with the ability to migrate within the 3D fibrin matrix substantiates the assertion that the cells contained within the fragments are preserved by the mechanical process and that the microfragments are capable of generating viable cells. Furthermore, the microfragment-conditioned medium was observed to maintain the viability of MSCs with a potency that was found to be superimposable with that of the medium containing 10% FBS. These assays confirm the viability of the fragments, indicating that adipose tissue fragments prepared with T-Grinder™ could be utilized to generate a culture medium devoid of xenogenic supplements. The biological activity of this medium would be supported by bioactive molecules produced by autologous cells, thereby enhancing the safety of the cell preparations for clinical applications.

A potential weakness of the present study is that our analysis does not allow for a direct assessment of the biological activity of microfat-derived cells by specific potency assays. To date, unambiguous procedures to determine the therapeutic potential of MSCs through these assays have not been validated, although they are widely discussed in the scientific literature. In a recent paper by Guest et al. [15], a number of parameters have been proposed for the standardization of procedures for the preparation of MSCs in veterinary regenerative medicine. In particular, in vitro immunosuppression assays are proposed to verify the immunomodulatory activity of MSCs, which is considered essential in different clinical contexts. In the present work, a direct comparison between the two cell populations using an immunosuppression assay was not performed. Instead, the expression of a panel of genes encoding proteins involved in the biological and therapeutic effects of MSCs was evaluated [41,44]. The panel included a set of genes involved in the biological and therapeutic effects of MSCs. TSG-6 and IL-1-Ra contribute to the anti-inflammatory and immunomodulating activity of MSCs [44,45]. The SDF-1/CXCR4 axis is a key player in the homing of MSCs to damaged tissues [46,47]. STC-1 has a protective role against reactive oxygen species (ROS) as well as potential anti-inflammatory activity [48,49]. COX-2 and PGES are involved in the synthesis of PGE2. PGE2 secreted by MSCs is a regulator of macrophage polarization toward the anti-inflammatory M2 phenotype that protects against exacerbated inflammation [45,50]. CCL-2 promotes angiogenesis and wound healing and is a key factor in the migration of MSCs [51,52]. With regard to the expression of these genes, no differences in expression were highlighted for any of the genes analyzed, suggesting a functional correspondence between the MSCs obtained from the two preparation methods. We are aware that the features we have analyzed are only partially indicative of the therapeutic functions of these cells. To gain a more comprehensive understanding of their therapeutic potential, a more in-depth functional comparison is necessary.

In summation, our results provide proof-of-concept evidence that the presented device can obtain biologically active microfat from a bolus sample of perivisceral adipose tissue obtained during surgery. To assess the viability and general equivalence of MSCs derived from mechanical processing versus standard enzymatic digestion of canine adipose tissue and to evaluate the biological activity and characteristics of the two cell groups, only a limited statistical donor population is required. It is acknowledged that further evaluation of adipose tissue samples is necessary to validate the repeatability and reliability of the results obtained. Furthermore, a larger number of samples should form the basis for subsequent controlled studies dedicated to the evaluation of the clinical outcome of the proposed one-step regenerative therapy on a controlled and statistically significant cohort of selected patients.

The use of an adipose tissue bolus is an alternative to the use of liposuction to obtain subcutaneous adipose tissue. The feasibility of liposuction in the canine patient may be influenced by the size and nutritional status of the patient [38]. Collecting a bolus of 8–10 g of abdominal adipose tissue is indeed feasible in different patients, meaning that this procedure can be performed on both small and large size dogs [38]. Further objectives would be to test the T-Grinder™ for the fragmentation of other tissue types such as subcutaneous fat, adjusting the protocol for different final tissue volumes and textures. As shown in the video reported in Supplementary Materials (Video S1), the overall procedure is rapid and practical, which allows for the use of the device by the surgeon without the need for external operators and the maintenance of sterility until the final product is ready to use. The collection, preparation and clinical application could be performed in the same surgery, thus avoiding multiple sedations or time-consuming delays for the patient.

The device has been designed with the specific goal of processing fat tissue with a reduced application of shear stress, which is the dominant stress applied to tissues and cells when they are extruded through a small striction, such as, for example, through small lumen tubes or sieves [53,54]. Shear stress can induce modifications in cell behavior by triggering mechano-sensing pathways. Rearrangements in cell organization, changes in gene expression, induction of apoptosis and tissue fibrosis have been demonstrated in various cell types [54]. Gene expression modifications have also been observed in canine bone-marrow-derived MSCs following the application of different shear stresses [55]. A recent review by You et al. [56] has evaluated different mechanical approaches to isolating adipose-tissue-derived stromal cells, suggesting that excessive mechanical stress leads to reduced stromal cell numbers and decreased cell viability. Furthermore, shear stress modulates pluripotency marker genes associated with the regenerative potential. In the protocol proposed here, controlled shear stress is achieved by a sharp fragmenting mechanism and microfat extrusion through relatively large pores (2 mm), which make the shear stress controlled, reproducible and not so intense as to cause measurable cell damage. The device is suitable for tissue amounts ranging from 6 g to 20 g and allows for the extraction of 4 mL to 15 mL of product. The microfat can be administered directly to the patient or used to expand in vitro MSCs for subsequent applications. Recently, Favaretto et al. [57] demonstrated that microfragmented adipose tissue can be stored for long periods of time, preserving the cellular composition and the ability to isolate and expand viable MSCs. A preliminary morphological characterization of the microfat obtained with T-Grinder™ (see Supplementary Materials File S1; Figures S1 and S2) supports the hypothesis that cryopreservation of the microfat is possible. Further studies are required to demonstrate the biological characteristics of cryopreserved microfat. Nevertheless, the device could be used to prepare microfragmented fat for immediate use or to be cryopreserved for future use. These features make the T-Grinder™ an interesting option for clinical applications, as it is possible to obtain both tissue (microfragmented fat) and cellular preparations suitable for repeated applications from a single tissue sampling.

## 4. Materials and Methods

Media, supplements, and other cell culture reagents used for cell culture were from Gibco (ThermoFisher Scientific Inc., Waltham, MA, USA) unless otherwise specified. Plastic labware was from VWR (Avantor, Radnor, OR, USA). A detailed list of cell culture reagents is provided in Supplementary File S2.

### 4.1. Ethics Statement

All the biological material used in the present study was collected in compliance with ethical regulations and animal welfare. The adipose tissue was provided from waste material following elective surgeries performed at the Veterinary Teaching Hospital (OVUD, University of Parma, Department of Veterinary Medicine). Each owner signed a written consent before the surgery. The collection of biological material for the isolation and expansion of MSCs was approved by the Ethics Committee of the University of Parma (OPBA protocol: n. 05/CESA/2024; approval date: 29 February 2024 and n.103/OPBA/2017; approval date: 11 April 2017).

### 4.2. Adipose Tissue Harvesting and Processing

Visceral abdominal adipose tissue (mean 19 ± 4.6) was collected from 4 healthy donors (2 males and 2 females, mean age 4.5 ± 2.64; mean weight 26.05 ± 10.75) during elective surgeries. The patients’ details are shown in Table 2.

**Figure 11:**
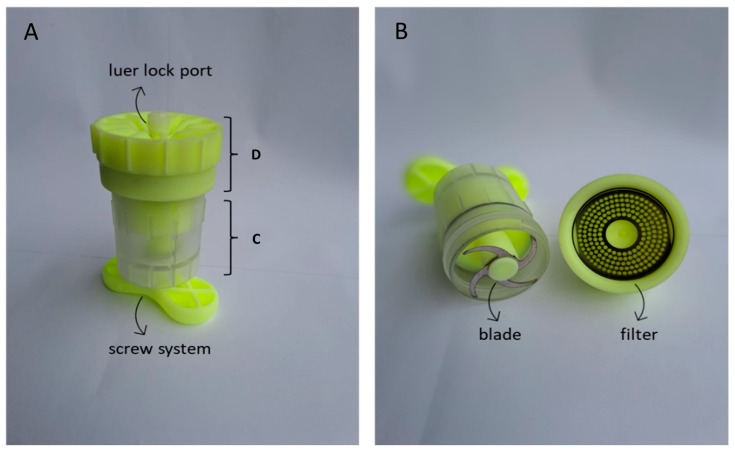
The T-Grinder^TM^ device composition. (**A**) External view of the device with the screw system, the adipose tissue collection chamber (D), the locking system (C), and the luer lock port. (**B**) Internal details of the blade and filter between the collection chamber and the locking system.

Biopsies were collected into 50 mL sterile Falcon tubes containing DMEM with penicillin (50 U/mL) and streptomycin (20 µg/mL) and immediately transported to the laboratory. Part of the tissue was processed to obtain microfragmented adipose tissue (mFAT), using the sterile T-Grinder™ device illustrated in Figure 11. In detail, 10 g of fat tissue was washed twice with phosphate-buffered saline pH 7.4 supplemented with penicillin (50 U/mL) and streptomycin (20 µg/mL) (cPBS) and with 70% ethanol solution, then placed in the collection chamber (Figure 11A(C)). The device was closed using the lock system (Figure 11A(D)), and the screw was tightened clockwise to push the adipose tissue through the blade and sieve system (2 mm pores) (Figure 11B). Finally, a luer lock syringe was connected to the port to collect the fragmented tissue. The adipose tissue was subjected to a single passage through the blade–filter system. A video tutorial of the overall procedure is available in the Supplementary Materials (Video S1).

An aliquot of the starting adipose tissue was washed and fragmented using a sterile scalpel under a cell culture hood and used as a control group (fFAT). For each donor, 3 g of the obtained mFAT or fFAT were used for the following experiments. When needed, adipose fragments were cryopreserved at −80° using a freezing medium consisting of 50% (*v*/*v*) FBS, 10% (*v*/*v*) dimethyl sulfoxide and 40% (*v*/*v*) DMEM with antibiotics.

### 4.3. Histological Analysis

Processed and original tissue samples were fixed with 10% neutral buffered formalin solution, dehydrated in a graded series of ethanol to absolute alcohol, cleared with xylene and embedded in paraffin. Five-micrometer-thin sections were stained with hematoxylin and eosin and by Masson trichrome staining [58]. Images were acquired with a Nikon Eclipse E800 microscope at various magnifications.

### 4.4. Cell Expansion and Count

All samples of mFAT or fFAT were incubated with 0.1% *w*/*v* collagenase type I prepared in low-glucose DMEM (Dulbecco’s Modified Eagle’s Medium) supplemented with 50 U/mL penicillin, 20 µg/mL streptomycin and 2.5 µg/mL amphotericin B at a ratio of 5 mL/g of minced tissue. Enzymatic digestion was carried out in a water bath at 37 °C with mild agitation, which was interrupted once complete disaggregation of the fragments had occurred (45 min for fFAT and 15 min for mFAT). The cell suspension was then diluted with mDMEM (maintenance medium with 10% fetal bovine serum and antibiotics), filtered through a nylon filter (mesh 100 µm; Millipore SIGMA, Burlington, VT, USA), and centrifuged at 300× *g* per 10 min. The cell pellet was resuspended in 3 mL of mDMEM and aliquoted into three T25 culture flasks. The cells were maintained in an incubator at 37 °C in a 5% CO_2_ atmosphere, with the medium renewed every 72 h. When the cells reached about 80% confluency, they were detached using 0.05% Trypsin-EDTA in cPBS and counted. Cells were then seeded in triplicate into 6-well plates (6000/cm^2^) and then expanded, trypsinized and counted as described below (Section 4.5) until passage 3. An aliquot of cells was cryopreserved in liquid nitrogen in DMEM supplemented with 50% FBS, 10% DMSO and antibiotics when needed.

### 4.5. Determination of Cell-Doubling Time and Cell-Doubling Number

To determine the cell-doubling number (CDn) and cell-doubling time (cDT), cells derived from the initial seeding of mFAT or fFAT were seeded in triplicate into 6-well plates at a density of 6000 cells/cm^2^ and maintained at 37 °C and 5% CO_2_ in cDMEM for 96 h. Cells were detached with 0.05% trypsin–EDTA and counted using a Burker camera following incubation with Trypan blue (Trypan Blue Solution, 0.4% Gibco™, Thermo Fisher Scientific). Following trypsinization, 0.2 mL of the cell suspension was mixed with 0.5 mL of 0.4% Trypan blue and 0.3 mL of PBS. After 5 min, an aliquot of the cell suspension was transferred to the chambers of the Burker camera. Five different 1 mm squares were counted per chamber. Blue-stained cells were considered non-viable cells. The number of viable cells per mL was calculated as follows: average count per square × dilution factor × 10^4^. Two different researchers repeated the count.

For P2 and P3, the cell-doubling number (CDn) and cell-doubling time (DT) were calculated as suggested by Vidal et al. [59] and Roth [60]. The two parameters were evaluated as follows:

CD = ln (Nf/Ni)/ln2,

DT = CT/CDn,

where CT is the cell culture time, Nf is the final number of cells and Ni is the initial number of cells.

### 4.6. Ad-MSC Differentiation

To assess the differentiation ability of ad-MSCs derived from fresh mFAT, cells at P3 were seeded into six-well plates at a density of 6000 cells/cm^2^ in complete medium. When at 80% confluency, the cells were treated as follows:

− Adipogenic differentiation: Cells were treated with adipogenic differentiation media according to the manufacturer’s instructions (StemPro Adipogenesis Differentiation Kit; Fisher Scientific, Hampton, NH, USA). The medium was changed every 2–3 days. After 21 days, the cells were fixed with 70% ethanol, and lipid vacuoles were visualized with Oil Red O staining [61].
− Osteogenic differentiation: Cells were treated with osteogenic induction medium composed of complete medium supplemented with 100 nM dexamethasone, 10 mM glycerophosphate and 0.250 mM ascorbic acid. The medium was changed every 2–3 days. After 21 days, the cells were fixed with 1% paraformaldehyde, and extracellular calcified matrix deposition was visualized by Alizarin Red staining [61].
− Chondrogenic differentiation: Cells were treated with chondrogenic differentiation media according to the manufacturer’s instructions (StemPro Chondrogenesis Differentiation Kit; Fisher Scientific, Hampton, NH, USA). The medium was changed every 2–3 days. After 21 days, the cells were fixed with 4% formaldehyde, and chondrogenic proteins were visualized by Alcian Blue staining [61].

### 4.7. Evaluation of the Viability of Fat Microfragments

To evaluate the viability, microfragments for each preparation were cultured in 35 mm Petri dishes inside a 3D matrix prepared by mixing 30% mDMEM, 50% PPP and 10% (*v*/*v*) calcium gluconate 100 mg/mL (S.A.L.F., Bergamo, Italy). The final volume of the 3D fibrin-based matrix was 2 mL. Fifteen fat fragments were embedded into the matrix immediately before gelation, which was initiated by calcium gluconate supplementation. After the gelation was complete, the 3D gel was layered with 3 mL of serum-free culture medium. The medium was changed every three days until day 10, when vital microfragments were counted. Microfragments that highlighted the escape of cells and their diffusion into the surrounding environment were considered vital.

### 4.8. Gene Expression Analysis

The gene expression analysis of a panel of genes involved in the inflammatory response of MSCs (Table 3) was investigated in fresh mFAT-derived MSCs and compared with control cells as described in Andreoli et al. [41]. Total RNA was extracted from P3 MSC pellets (2 × 10^6^ cells) using NucleoSpin RNA II (Macherey-Nagel GmbH, Duren, Germany). cDNA was synthesized from 2 μg of RNA in reverse transcription reactions using the High-capacity cDNA reverse transcription kit (Applied Biosystems, Waltham, MA, USA). The qPCR assessment was performed with a 20 µL reaction volume consisting of cDNA transcripts, AceQ^®^ Universal SYBR Green qPCR Master Mix (Vazyme Biotech Co., Ltd., Nanjing, China) and the primer pairs (Eurofins Genomics, Ebersberg, Germany) shown in Table 2. All the primer pairs have been previously validated [41]. GAPDH was used as a housekeeping gene. The thermocycler program (Applied Biosystem QuantStudio1^TM^ Design and Analysis Software v1.5.2; ThermoFisher Scientific, Whaltam, MA, USA) was set as follows: initial pre-denaturation at 95 °C for 1 min, followed by 40 consecutive cycles consisting of denaturation at 95 °C, annealing of primers at 57 °C, elongation at 72 °C and, finally, a further extension at 60 °C.

### 4.9. Flow Cytometry

Cells at P3 were harvested from flasks using 0.01% *v*/*v* of TrypLE Express. Recovered viable cells were counted and resuspended in FACS flow reagent before being aliquoted into flow cytometry tubes. The surface markers used for the flow cytometric analysis are those commonly used in dogs for the characterization of MSCs, i.e., CD90, CD29 and CD44 as markers with positive expression and CD45, CD14 and MHCII as markers with negative expression. CD146 and CD31 were added to verify the presence of pericytes and/or endothelial cells. The antibodies used, listed in detail in Table 4, have been validated for canine species by the manufacturer.

Each antibody was diluted in PBS and added to the mix according to the manufacturer’s instructions. The labelling was performed on 100 µL of sample containing 1 × 10^6^ cells with a 15 min incubation in the dark at room temperature. Samples were washed and resuspended in FACS flow solution for acquisition using a FACS CantoII flow cytometer equipped with 2 lasers at 488 nm and 640 nm. Data analysis was performed with the BD FACSDiva^TM^ v8.0 software and Kaluza 2.1 (Beckman Coulter, Brea, CA, USA). Unstained cells were used as a control to detect autofluorescence.

### 4.10. Effects of Microfat-Conditioned Medium on Cell Vitality

To evaluate the effects of conditioned medium (CM) prepared from the microfragmented fat on MSC vitality, 1.5 g of the preparation obtained from the T-Grinder was added to 6 mL of serum-free DMEM containing antibiotics and incubated in T25 flasks for 3 days at 37 °C in a 5% CO_2_ atmosphere. The medium was then collected and replaced with fresh serum-free DMEM, collected after an additional 3 days of culture. The CM medium was then centrifuged at 900× *g* for 20 min to remove cell debris and then filtered through 25 µm mesh filters. To evaluate the effects of microfat CM on cell vitality by the MTT assay, adipose-tissue-derived MSCs were seeded into a 96-well plate at a density of 6000 cells/well (*n* = 6). The CM’s effect on cell vitality was evaluated following 10% and 50% dilution in serum-free medium or without dilution. Serum-free DMEM and DMEM supplemented with 10% FBS were used as references. The final volume was 100 µL/well. After 48 h of culture, 20 µL of a 5 mg/mL solution of MTT in phosphate-buffered saline (PBS), pH 7.4, was added. After 4 h, the formazan salts were solubilized by adding 100 µL of 10% SDS in 0.01 M HCl. Following overnight incubation, the formazan absorbance at 570 nm was recorded by a Victor Nivo spectrophotometer (Perkin Elmer; Groningen, the Netherlands). The experiment was performed with 6 different microfat preparations.

### 4.11. Statistical Analysis

Before performing statistical analyses, all the datasets were assessed for normality using the Shapiro–Wilk test, which confirmed that the data were normally distributed and thus suitable for parametric testing. To analyze the RT-PCR data related to gene expression, a two-way ANOVA was used to evaluate the effects of treatment and animal on the cellular population variability, including their interaction. This analysis enabled us to determine how these factors contributed to the observed differences among cell populations. For assessing the impact of microfat-conditioned medium on cell vitality, a one-way ANOVA was conducted to compare the means across multiple groups and to identify statistically significant differences. When significant differences (*p* < 0.05) were detected, Tukey’s Honestly Significant Difference (HSD) test was employed for post hoc comparisons. Tukey’s HSD test was selected due to its effectiveness in handling normally distributed data and its conservative nature in reducing false positives. Additionally, for cell counts and flow cytometry data, the Student’s t-test was utilized to compare differences between the two groups. All statistical analyses were carried out using Prism 8 (GraphPad, San Diego, CA, USA) and Jamovi (Version 2.5, https://www.jamovi.org, access date 10 September 2024).

## 5. Conclusions

The use of devices for the preparation of microfragmented fat suitable for immediate therapeutic application represents an interesting operational possibility for veterinary regenerative medicine. The device described in the present study, the T-Grinder^TM^, allows a ready-to-use microfat to be obtained from a 6–20 g bolus of perivisceral adipose tissue. The microfat obtained allows for the isolation of MSCs with phenotypic characteristics and replicative and differentiation capacities that are equivalent to those observed for MSCs isolated by enzymatic techniques. When cultured in a 3D matrix, the microfragments show cellular outgrowth, demonstrating their viability. Moreover, conditioned medium prepared from microfragmented fat supports cell viability when used as a substitute for fetal bovine serum, suggesting its potential use in the preparation of autologous MSCs for clinical applications.

## Figures and Tables

**Table 1:** Percentages and standard deviations of cell populations (*n* = 3) prepared using the two methods (enzymatic and mechanical) that were positive for the tested markers.

Marker	Enzymatic Method	Mechanical Method
CD29	87.78 ± 10.50	88.37 ± 9.96
CD90	99.73 ± 0.33	99.52 ± 0.71
CD44	82.91 ± 5.87	79.41 ± 10.13
CD45	0.02 ± 0.04	0.02 ± 0.04
CD14	0.05 ± 0.05	0.15 ± 0.25
MHC II	0.06 ± 0.11	0.17 ± 0.30
CD31	0.12 ± 0.01	0.30 ± 0.34
CD146	0.76 ± 1.26	5.91 ± 10.18

**Table 2:** Canine donors’ details in terms of breed, sex, age and weight.

Dog	Breed	Sex	Age (years)	Weight (kg)
1	Setter	M	2	15
2	Labrador retriever	M	8	39
3	Australian shepherd	F	3	20
4	Labrador retriever	F	5	30

**Table 3:** List of primers used for gene expression analysis in the canine MSCs.

Gene	Primer Sequence	Accession Number Amplicon Size
*TSG-6*	Fw: AATCGGATTTCACGTCTGCG	XM_533354.7
	Rv: CACCACACTCCTTTGCATGT	182 bp
*SDF-1*	Fw: GCCGATTCTTCGAGAGCCAC	NM_001308461.1
	Rv: TCTGCCATACGCTGTTAGCTT	240 bp
*IL-1-RA*	Fw: GAAGAGACCTTGCAGGATGC	AF216526
	Rv: GACGGGCACCACATCTAACT	141 bp
*STC-1*	Fw: CACTTCTCCAACAGATACT	XM_543238
	Rv: CATGTTGGGCCCAATTTTC	110 bp
*COX-2*	Fw: GATCATAAGCGAGGACCAGCTTTC	NM_001003354.1
	Rv: GGCGCAGTTTATGTTGTCTATCCA	100 bp
*PGES*	Fw: GTATTGCCGGAGTGACCAGGA	NM_001122854.1
	Rv: AGTGCATCTGGGCGATGAAAG	136 bp
*CCL-2*	Fw: TCCTCTGCCTGCTGCTCATAG	NM_001003297.1
	Rv: GCAGCAGGTGACTGGAGAAATAA	86 bp
*CXCR-4*	Fw: GAGCGGTTACCATGGAAGAG	NM_001048026.1
	Rv: CGGTTGAAGTGAGCATTTTCC	108 bp
*GAPDH*	Fw: GATGGGCGTGAACCATGAGA	NM_001003142.2
	Rv: AGTGGTCATGGATGACTTTGGCTA	107 bp

**Table 4:** List of antibodies used for the cytofluorimetric analysis.

Antibody	Producer	Code
CD14 monoclonal antibody (Tuk4) FITC	ThermoFisher Scientific (Carlsbad, CA 92008, USA)	MA1-82074
CD29 anti-human antibody (TS2/16) PE	BioLegend (San Diego, CA, USA)	303004
CD90 monoclonal antibody (YKIX337.217) PE	ThermoFisher Scientific (Carlsbad, CA 92008, USA)	12-5900-42
CD44 monoclonal antibody (YKIX337.8) FITC	ThermoFisher Scientific (Carlsbad, CA 92008, USA)	11-5440-42
CD45 monoclonal antibody (YKIX716.13) PE	Biorad (Hercules, CA, USA)	MCA1042PE
CD146 monoclonal antibody (P1H12) FITC	ThermoFisher Scientific (Carlsbad, CA 92008, USA)	11-1469-42
CD31 polyclonal antibody PE	Bioss Antibodies (Woburn, MA, USA)	bs-0468R-PE
Rat anti-dog MHC Class II monomorphic (YKIX334.2) FITC	Biorad (Hercules, CA, USA)	MCA1044F

## Data Availability

Data are contained within the article and Supplementary Materials.

## Supplementary Materials

The supporting information can be downloaded at: https://www.mdpi.com/article/10.3390/ijms252111854/s1.
