# Supplementary material for: Evaluation of a Novel Mechanical Device for the Production of Microfragmented Adipose Tissue for Veterinary Regenerative Medicine: A Proof-of-Concept
Source: Int J Mol Sci. 2024 Nov 4;25(21):11854. doi: 10.3390/ijms252111854 (PMC11546731; doi:10.3390/ijms252111854)
Supplement: Supplementary file 1 [file ijms-25-11854-s001.zip › Supplementary File S1.pdf]

Supplementary Materials

<u>3D culture of microfragmented fat following cryo-preservation.</u>

Adipose fragments obtained with manual fragmentation (using a sterile scalpel) and those obtained using T-Grinder™ device were cryopreserved at -80° using a freezing medium consisting of 50% (v/v) FBS, 10% (v/v) dimethyl sulfoxide, and 40% (v/v) DMEM with antibiotics. After 30 days, adipose fragments were thawed in a thermostatically controlled bath at 37°C and seeded in 35 mm Petri dishes, inside a 3D matrix prepared by mixing 30% DMEM with antibiotics, 50% platelet-poor plasma, and 10% (v/v) calcium gluconate 100 mg/mL (S.A.L.F., Italy). The final volume of the 3D fibrin-based matrix was 2 mL. The medium was changed every three days until day 10 when vital micro-fragments were counted. Microfragments that highlighted the outgrowth of cells and their migration into the surrounding environment were considered vital.

After 10 days of culture, the 3D culture of micro-fragments demonstrated an active outgrowth of cells (**Figure S1 and Figure S2**) gradually expanding inside the matrix itself in 100% of the seeded fragments, with no differences between the preparations (n=4).

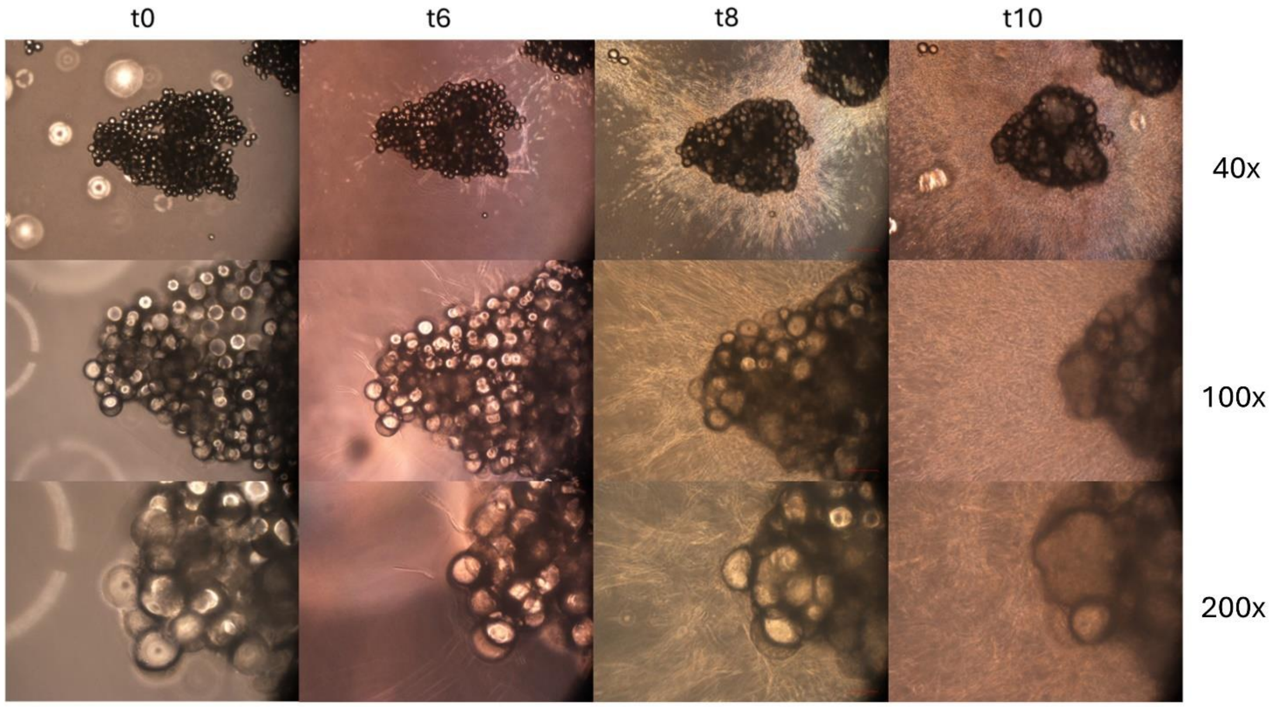

Figure S1. 3D culture of frozen adipose fragments obtained with manual fragmentation, seeded in 3D matrix at 0 (t0), 6 (t6), 8 (t8), 10 (t10) days of culture.

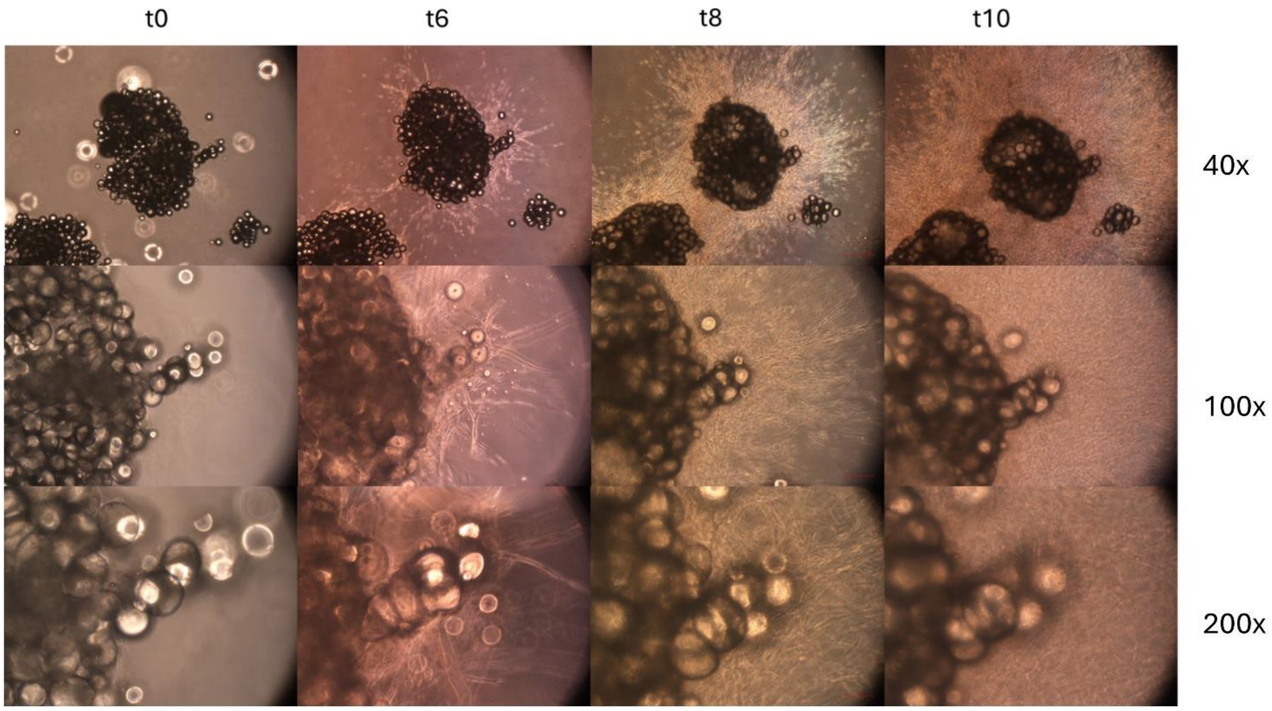

Figure S2. 3D culture of frozen adipose fragments obtained with mechanical fragmentation, seeded in 3D matrix at 0 (t0), 6 (t6), 8 (t8), 10 (t10) days of culture.
