# Supplementary material for: Evaluation of a Novel Mechanical Device for the Production of Microfragmented Adipose Tissue for Veterinary Regenerative Medicine: A Proof-of-Concept
Source: Int J Mol Sci. 2024 Nov 4;25(21):11854. doi: 10.3390/ijms252111854 (PMC11546731; doi:10.3390/ijms252111854)
Supplement: Supplementary file 1 [file ijms-25-11854-s001.zip › Supplementary File S2 (material).pdf]

REAGENTS AND MATERIALS

- Alcian blue (Sigma-Aldrich, Inc, St. Louis, USA)
- Amphotericin (Thermo Fisher Scientific Inc, Whaltam, USA)
- Ascorbic acid (Sigma-Aldrich, Inc, St. Louis, USA)
- Calcium gluconate 1000 mg/10 ml (SALF, Bergamo, Italy)
- Dexamethasone (Sigma-Aldrich, Inc, St. Louis, USA)
- DMEM - Dulbecco's Modified Eagle's Medium (Thermo Fisher Scientific Inc, Whaltam, USA)
- DMSO dimethyl sulfoxide (Thermo Fisher Scientific Inc, Whaltam, USA)
- Ethidium bromide (Invitrogen DNA Ladder, Thermo Fisher Scientific – US)
- Fetal Bovine Serum- FBS (Thermo Fisher Scientific Inc, Whaltam, USA)
- Glycerophosphate (Sigma-Aldrich, Inc, St. Louis, USA)
- MTT (3- (4,5-Dimethylthiazol-2 -yl) -2,5-diphenyltetrazolium bromide) (VWR Scientific Avantor, Radnor, USA)
- Oil Red O stain (Sigma-Aldrich, Inc, St. Louis, USA)
- Paraformaldehyde (Bio Optica, Milan, Italy)
- Penicillin (Thermo Fisher Scientific Inc, Whaltam, USA)
- Physiological saline solution 0,9% (SALF, Bergamo, Italy)
- Ringer Lactate Solution (RLS) (SALF, Bergamo, Italy)
- RT-PCR PRIMERS (Macrogen Europe Meibergdreef 57 1105 BA, Amsterdam, The Netherlands) and (Eurofins Genomics, Germany GmbH Anzinger Str. 7a 85560 Ebersberg, Germany)
- SDS in 0.01 M HCl (Thermo Fisher Scientific Inc, Whaltam, USA)
- Sodium citrate (Thermo Fisher Scientific Inc, Whaltam, USA)
- Streptomycin (Thermo Fisher Scientific Inc, Whaltam, USA)
- Trypan Blue Solution 0.4% (Gibco™, Thermo Fisher Scientific)
- Trypsin-EDTA (Thermo Fisher Scientific Inc, Whaltam, USA)
- Von Kossa staining (Bio Optica, Milan, Italy)
- AceQ® Universal SYBR Green qPCR Master Mix (Vazyme biotech co., Nanjing, PRC)
- Applied Biosystems™ High-Capacity cDNA Reverse Transcription kit (Applied Biosystems, Cheshire, UK)
- Bestaq™ DNA Polymerase Kit (Applied Biological Materials Inc.,|Richmond, Canada)
- Labware was from VWR (Avantor, Radnor, USA)
- NucleoSpin® RNA kit (MACHEREY-NAGEL, GmbH & Co. KG)
- StemPro Adipogenesis Differentiation Kit (Gibco Thermo Fisher Scientific Inc, Whaltam, USA)
- StemPro Chondrogenic Differentiation kit (Gibco Thermo Fisher Scientific Inc, Whaltam, USA)
- TrypLE Express (Thermo Fisher Scientific Inc, Whaltam, USA)
